# Stress and Auditory Responses of the Otophysan Fish, *Cyprinella venusta*, to Road Traffic Noise

**DOI:** 10.1371/journal.pone.0137290

**Published:** 2015-09-23

**Authors:** Jenna A. Crovo, Mary T. Mendonça, Daniel E. Holt, Carol E. Johnston

**Affiliations:** 1 Fish Biodiversity Lab, School of Fisheries, Aquaculture, and Aquatic Sciences, College of Agriculture, Auburn University, Auburn, Alabama, United States of America; 2 Department of Biological Sciences, College of Sciences and Mathematics, Auburn University, Auburn, Alabama, United States of America; 3 Department of Earth and Space Sciences, College of Letters and Sciences, Columbus State University, Columbus, Georgia, United States of America; University of Auckland, NEW ZEALAND

## Abstract

Noise pollution from anthropogenic sources is an increasingly problematic challenge faced by many taxa, including fishes. Recent studies demonstrate that road traffic noise propagates effectively from bridge crossings into surrounding freshwater ecosystems; yet, its effect on the stress response and auditory function of freshwater stream fishes is unexamined. The blacktail shiner (*Cyprinella venusta*) was used as a model to investigate the degree to which traffic noise impacts stress and hearing in exposed fishes. Fish were exposed to an underwater recording of traffic noise played at approximately 140 dB re 1 μPa. Waterborne cortisol samples were collected and quantified using enzyme immunoassay (EIA). Auditory thresholds were assessed in control and traffic exposed groups by measuring auditory evoked potentials (AEPs). After acute exposure to traffic noise, fish exhibited a significant elevation in cortisol levels. Individuals exposed to 2 hours of traffic noise playback had elevated hearing thresholds at 300 and 400 Hz, corresponding to the most sensitive bandwidth for this species.

## Introduction

Acoustic signaling is an important mode of communication for numerous taxa, including fishes [1, 2]. At present, sound production is documented in over 800 species of fish representing 109 families [3]. In many fishes, sound production accompanies numerous crucial behaviors including: courtship, spawning, agonistic interactions, and competitive feeding [4–6]. Many species utilizing acoustic cues, including those in the series Otophysi, also possess ancillary hearing adaptations to detect these signals. Otophysan fishes constitute approximately 64% of all known freshwater fishes [7].

Currently, there is a growing volume of literature documenting the deleterious effects of anthropogenic noise on acoustic communication in the natural world [8–10]; although one study with birds found no effect of road noise on stress hormones [11]. Noise generated from human-related activities, such as transportation networks, commercial shipping, seismic exploration, resource extraction, and urbanization presents a unique challenge to organisms in both terrestrial and aquatic environments [12–14]. Anthropogenic noise often has frequencies similar to those utilized by vertebrates that communicate acoustically, effectively masking communication networks [13, 15]. Furthermore, in contrast to natural sources of ambient noise in the environment, the comparatively recent presence of anthropogenic noise from an expanding human population has not provided adequate time for acoustic signal evolution.

In addition to disrupting communication networks, chronic anthropogenic noise has detrimental effects on the physiology and behavior of numerous exposed species [16–18]. In fishes, noise exposure compromises fitness related behaviors, such as foraging and antipredator responses [19, 20]. Exposed fishes also experience changes in auditory and neuroendocrine function. Several studies examined the effect of boat noise on the auditory thresholds of fishes [21–24]. For soniferous otophysans, decreased auditory sensitivity is particularly harmful because it may reduce the capacity to detect acoustic signals from conspecifics. Hearing loss for these species would be deleterious during the mating season as acoustic signals are a crucial component of courtship [2, 4]. Limited auditory function may be further compounded by the masking effects of traffic noise [15]. Consequently, otophysans are especially vulnerable to the growing omnipresence of anthropogenic noise. In conjunction with a shift in auditory thresholds, boat noise elicits an increase in the stress hormone, cortisol, in several freshwater fishes [8].

While noise generated from aquatic activities pose a clear threat to fishes, research demonstrates that road traffic noise from bridge crossings propagates a significant distance into freshwater streams [15]. The effect of this noise on the stress response and auditory thresholds of exposed freshwater fishes is unexplored. As road networks continue to expand globally, it is imperative to understand how otophysan fishes, which dominate freshwater systems, are affected by present traffic noise levels [25, 26]. To elucidate the effects of road traffic noise, we designed a series of manipulative experiments to measure changes in cortisol levels and auditory thresholds of a model soniferous otophysan, the blacktail shiner (*Cyprinella venusta*), during exposure to an underwater recording of road traffic noise.

## Materials and Methods

### Fish Collection and Maintenance

We collected *C*. *venusta* from Little Uchee Creek in Moffits Mill located in Lee County, Alabama (32. 549244°N; -85.278513°W) using 10 ft seine nets. The permit to collect *C*. *venusta* at this location was issued by the Alabama Department of Conservation and Natural Resources (ACDNR). Immediately after collection, fish were transported to the Fish Biodiversity Lab in coolers containing creek water. An aerator was also provided for each cooler. Fish were housed in 20 L aquaria with gravel substrate and allowed to acclimate 24 hours prior to testing. Fish were maintained at ambient light and temperature conditions. Fish were fed bloodworm (*Chironomid sp*.) larvae daily. The methods used for this project were approved by the Animal Care and Use Committee at Auburn University (protocol number 2012–2016: 2333).

### Traffic Noise Acquisition

The road traffic noise used for the stress and auditory experiments was recorded at a beam bridge crossing in Macon County, Alabama in March 2010 as part of a traffic noise propagation study. A hydrophone (Hi-tech HTI-96-MIN, sensitivity—164.4 re 1V/μPa, frequency response: 0.002–30 kHz) connected to a digital recorder (Marantz PMD 661) sampling at a rate of 44.1 kHz was used to record traffic. The hydrophone was positioned 8 cm off the stream bed and 3 m downstream from the bridge piling. The hydrophone was attached to the end of a PVC pipe. This pipe was secured between two submerged sandbags; the sandbags were placed downstream from the hydrophone. Traffic was recorded for several minutes. The methods used to record the traffic noise used in this study are fully described in Holt and Johnston (2015).

### Waterborne Cortisol Collection

Waterborne cortisol samples were collected between 4 and 6:30 am during the months of February and March 2014. This test period was chosen because noise from passing vehicles could be detected in the test room, and there was minimal car traffic on the road next to the lab building at this time. Individual test fish (n = 7) were placed into a rectangular 700 mL glass collection dish containing 450 mL of dechlorinated water. Once in the collection dish, fish were partially submerged in a 200 L aquarium and positioned 6.5 cm away from an underwater speaker (UW—30, Universal Sound Inc., Oklahoma City, OK). The collection dishes were large enough so that fish could move freely to mitigate confinement stress. Each fish received the control and noise treatment on separate days; the treatment order was randomized. To control for diel fluctuations in cortisol levels, fish were tested at the same time of day for both treatments.

The control treatment consisted of a 30 min period of silence. During this treatment, nothing was played from the underwater speaker; the speaker was not connected to the playback system. The traffic treatment consisted of a traffic recording looped for 30 minutes; this recording was played at a volume of approximately 140 dB re 1 μPa. To determine the playback volume, a hydrophone was placed in a collection dish that was positioned 6.5 cm away from the underwater speaker. This hydrophone was connected to a digital recorder (Marantz PMD 661; sampling rate 44.1 kHz). The traffic recording was presented from a Dell laptop using Raven Pro 1.4 software (Cornell University, Ithaca, NY). The laptop was connected to a SLA1 studio amplifier (Applied Research and Technologies); the underwater speaker in the 200 L test aquaria was also connected to this amplifier. The traffic playback file recorded on the Marantz digital recorder was analyzed in Raven Pro 1.4 (Cornell University, Ithaca, NY). The peak dB levels were measured in 2 s intervals and averaged to estimate the approximate volume.

At the end of each trial, water samples were filtered immediately using primed C-18 cartridges (Sep-pak, Waters Technology Corporation, Milford, MA). Cartridges were stored at-80°C prior to running assays. Free cortisol was eluted from cartridges with two 2 mL washes of ethyl acetate and evaporated under nitrogen gas. The dried residues were resuspended in enzyme immunoassay (EIA) buffer. All samples were diluted (1:200) prior to being loaded into the plate. The plated was incubated and developed in accordance with the Cortisol EIA kit instructions (Cayman Chemical, Ann Arbor, MI). The cortisol kit was validated for use with *C*. *venusta* by achieving parallelism between the standard curve provided with the kit and a serially diluted sample of pooled *C*. *venusta* cortisol.

### Measuring Auditory Thresholds

**Fig 1:**
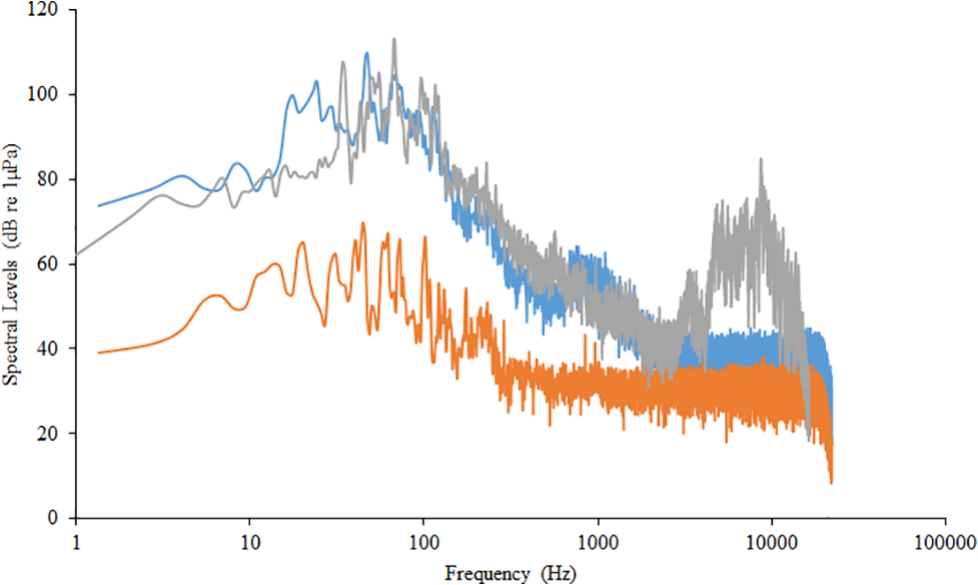
Spectral characteristics of acoustic stimuli. Power spectra for traffic playback (gray line) and control treatments (orange line). The power spectrum of the original traffic recording is provided for reference (blue line). Spectra were created with a resolution of 1.35 Hz.

The experimental design to assess the effect of traffic noise on auditory sensitivity was similar to the design used for cortisol collection. Individuals were exposed to the same traffic recording looped for a period of 2 hours at 140 dB re 1 μPa (n = 5). Individuals in the control treatment were exposed to a period of silence for 2 hours; no sounds were played from the speaker during the control treatment (n = 5). These trials were conducted in the same tank used for the cortisol collections. As these tests consisted of a longer exposure period, individuals were placed in a 10 L square tank. The distance between the edge of tank and the underwater speaker was 7 cm. Power spectra illustrating the spectral characteristics of the traffic playback and the ambient noise from the control treatment are presented in Fig 1. It is true that the glass walls of aquaria cause reverberation of the acoustic stimulus. The minimum resonant frequency that would create distortion in the experimental design we used was 6, 372 Hz, which is well above the hearing threshold of *C*. *venusta* [27].

Additionally, particle acceleration of the traffic noise was measured as the difference in RMS pressure between two hydrophones (a = 0.195). Acceleration was calculated using the following formula: a = –((p_1_—p_2_ / d) / ρ)). In this formula, p_1_—p_2_ is the pressure difference between the two hydrophones; d is the distance between the hydrophones; ρ is the density of freshwater (997.1 kg/m^3^). Particle acceleration measures from three orthogonal axes were combined into a single value using the equation: $a=\sqrt{x^{2}+y^{2}+z^{2}}$, where x, y, and z are the three axes. The degree of particle acceleration (m s^-2^) associated with the frequency tones presented during the AEP trials was calculated for each dB level using the same method [28]. The two recording hydrophones were placed 3 cm apart in the AEP test chamber; the midpoint between the hydrophones corresponded to the location of the fish’s head during a trial. Electrical noise from the underwater speaker prevented the calculation of particle accelerations below 95 dB. Consequently, the particle accelerations of tones between 80 and 100 dB were extrapolated using data from 105 to 150 dB. These methods are fully outlined in Holt and Johnston (2011).

Auditory sensitivity after each treatment was determined by measuring auditory evoked potentials (AEPs). Auditory thresholds were measured at 100, 200, 300, 400, 600, 800, and 1,000 Hz; these frequencies are within the hearing range of *C*. *venusta*. Each frequency was presented to individual fish from 70–155 dB in 5 dB increments, and 250 responses were averaged for each presentation. These tones were 10 ms in duration with a rise and fall time of 2 ms. The frequency tones were generated using Sig Gen software and hardware (Tucker Davis Technologies, Gainesville, FL) and played through an underwater speaker (UW—30, Universal Sound Inc., Oklahoma City, OK) located in the auditory test chamber (Tremetrics AR 9S Audiometric Booth). The test tank inside the chamber was a 79 cm section of PVC pipe that was capped at both ends with a 16.5 × 52 cm hole located in the top of the pipe. Water was filled to a depth of 23 cm. The underwater speaker was suspended 10 cm below the water’s surface and 39 cm away from the left side of the tank.

Prior to testing, frequency tones were calibrated using a hydrophone (Hi-Tech HTI-96-MIN, sensitivity-164.4 re 1V/μPa) and a GW GOS-6xxG dual trace oscilloscope. During calibration, the hydrophone was positioned where the recording electrode on the fish’s head would be located during a trial. For each tone, the peak voltage readings from the oscilloscope were converted to dB re 1 μPa, and these values were used to compile a normalization file. The normalization file was used to make adjustments to ensure that the sound pressure levels were correct. The corrected frequency tones were played to the fish using BioSig software and hardware (Tucker Davis Technologies, Gainesville, FL).

After each treatment, test fish were wrapped in gauze and restrained in a clay bed at a depth of 11 cm and 8 cm away from the underwater speaker (UW-30, Universal Sound Inc., Oklahoma City, OK). Auditory thresholds were measured as the potential difference between electrodes (Rochester Electro-Medical, Inc., Tampa, FL) inserted subcutaneously above the brainstem and in the caudal peduncle. A grounding electrode was inserted into the clay bed. Electrodes were insulated with fingernail polish except at the tip inserted into the fish. Electrodes fed into a Medusa RP2.1 pre-amplifier connecting to a RA16 base station processor feeding to the BioSig software (Tucker Davis Technologies, Gainesville, FL). Auditory traces were generated using BioSig software. Tones were also presented in opposite phases (90° and 270°), and the traces were averaged to cancel stimulus artifacts. Auditory thresholds were determined visually as the lowest sound level to elicit an AEP response. Audiograms generated from visually assessing thresholds are similar to audiograms produced using statistical methods [29].

## Results

**Fig 2:**
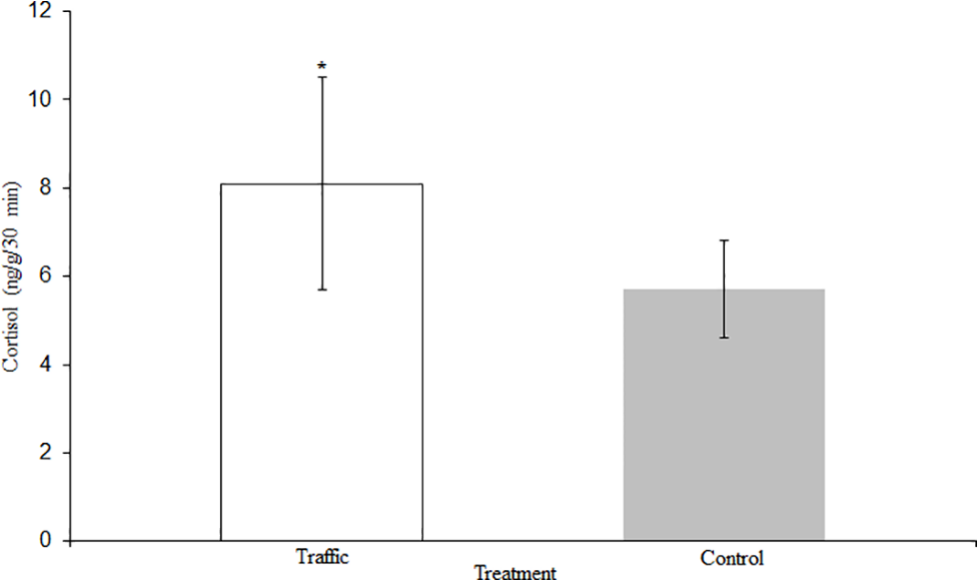
Waterborne Cortisol Levels. Cortisol levels for *C*.*venusta* exposed to the control and traffic treatments presented as the mean ± SE ng g^-1^ min^-1^. * denotes p < 0.05.

Cortisol levels were significantly elevated after traffic noise exposure (n = 7, F_1, 6_ = 6.546, p = 0.043). Individuals released 8.2 ± 2.3 ng g^-1^ 30 min^-1^ when exposed to traffic noise and 5.7 ± 1.1 ng g^-1^ 30 min^-1^ when exposed to the control treatment (Fig 2). These results equate to approximately a 44.0% change in the cortisol production of exposed *C*. *venusta*. The intra-assay coefficient of variation was 2.3%. The inter-assay coefficient of variation was not calculated because all of the samples were analyzed on one plate.

**Fig 3:**
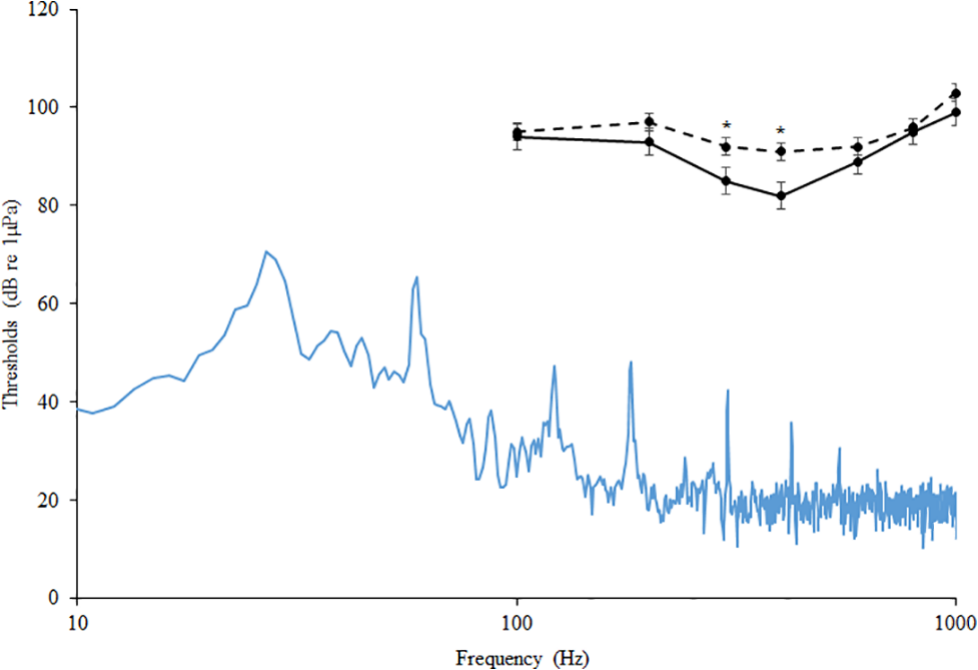
Pressure Audiogram. Auditory hearing thresholds for *C*. *venusta* exposed to control treatment (solid line) and traffic treatment (dashed line) presented as the mean SPL ± SE dB re 1 μPa. * denotes p < 0.05. The power spectrum of the background noise in the test chamber is represented by the blue line.

**Fig 4:**
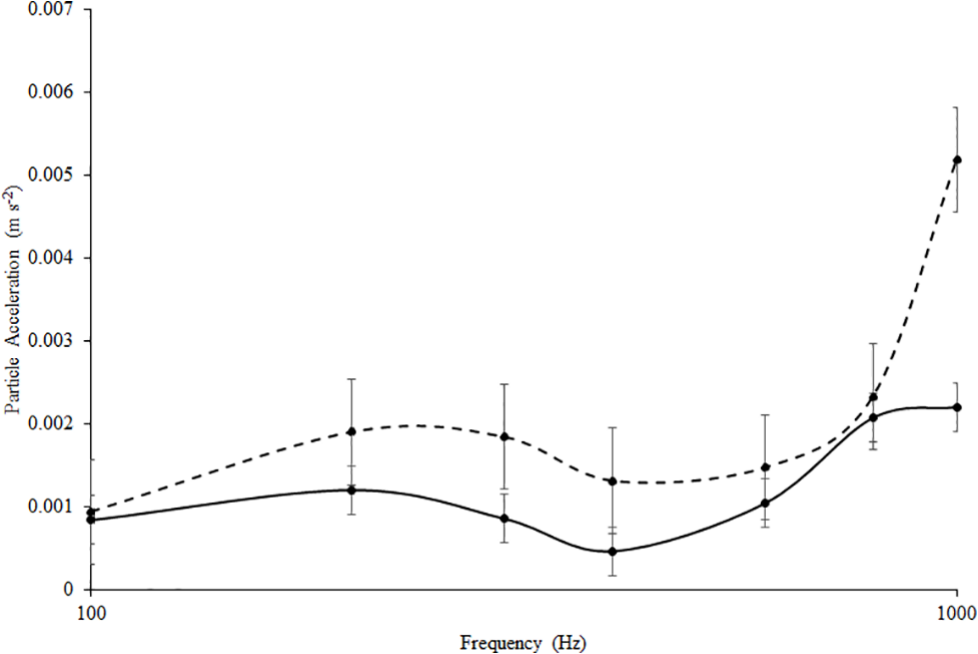
Particle Acceleration Audiogram. Auditory hearing thresholds for *C*. *venusta* exposed to control (solid line) and traffic treatment (dashed line) expressed as the mean acceleration ± SE m s^-2^.

The pressure audiogram generated from control individuals showed that the auditory thresholds of *C*. *venusta* were most sensitive at 300 and 400 Hz (n_control_ = 5). The auditory thresholds were 85 dB at 300 Hz and 82 at 400 Hz. After 2 hours of traffic playback, auditory thresholds were elevated at 300 and 400 Hz (n_traffic_ = 5). Auditory thresholds shifted upward significantly by 7 dB at 300 Hz (p = 0.020) and 9 dB at 400 Hz (p = 0.025). Auditory sensitivity at all other test frequencies remained virtually unchanged between the two groups (Fig 3). The particle acceleration audiogram also showed similar auditory sensitivity at 300 and 400 Hz for control individuals (Fig 4).

### Statistical Analysis

Waterborne cortisol levels for control and traffic treatments were compared using a one-way repeated ANOVA. Auditory thresholds were compared via a repeated one-way ANOVA on frequency and treatment. Data were tested for normality using the Kolmogorov-Smirnov test. An alpha criterion of 0.05 was used for all tests. All statistics were completed using SPSS version 22 (IBM SPSS Corporation, Chicago, IL).

## Discussion

The results reported in this study are consistent with the literature documenting the effects of anthropogenic noise on stress and hearing; however, this is the first study to investigate the effects of terrestrial traffic noise on an otophysan, freshwater stream fish [8, 14, 21]. The elevation in *C*. *venusta* cortisol during acute traffic noise exposure was likely influenced by a combination of the sound pressure level, temporal variability, and acoustic structure of road traffic (S1 Fig). The noise generated from road traffic fluctuates with respect to frequency and amplitude; noise levels also vacillate throughout the day, making it an irregular stressor. Intermittent acoustic stimuli, such as traffic noise, are suggested to have more prolonged effects on the stress response than constant stimuli [16–18]. Similar cortisol elevations were observed in fishes exposed to recordings of boat noise [8]. Our study investigated the acute effects of traffic exposure on the stress response of *C*. *venusta*; whether this species acclimatizes to traffic noise in the environment over time remains to be determined.

Auditory threshold shifts were greatest at 300 and 400 Hz—where *C*. *venusta* hearing was most sensitive. These results are of biological significance as the agonistic and courtship signals of this species have similar frequencies [15]. A potential mechanism explaining the observed threshold shifts is sensory hair cell damage from traffic noise exposure. Several studies show a correlation between noise exposure and the apoptosis of hair cells in fishes [30, 31]. It is noteworthy to recognize that fishes continue to add hair cells to auditory epithelia throughout ontogeny and have the capacity to regenerate lost cells after acoustic trauma [30, 32]. The time required to regain pre-exposure auditory thresholds, however, is dependent upon the intensity and duration of the noise [30]. In a study by Smith et al (2004), goldfish, *Carassius auratus*, exposed to 170 dB re 1μPa white noise for 24 hours experienced significant, albeit not complete, recovery after 18 days [33]. A similar study exposed *C*. *auratus* to 158 dB re 1μPa white noise for 24 hours; individuals recovered auditory thresholds after 3 days [34]. We did not establish the time required for *C*. *venusta* to regain auditory thresholds after traffic noise exposure; however, even a temporary period of reduced auditory function could be detrimental for vital behaviors such as conspecific communication and predator detection. Previous work demonstrates that *C*. *venusta* increases its signal amplitude under noisy conditions [35]. It is unknown if this behavioral plasticity is sustainable for long periods. Future work should investigate auditory shifts and recovery periods for a series of exposure levels to further understand the ecological effects of traffic noise in the environment.

There are caveats to investigating the impacts of traffic noise on fishes under laboratory conditions. The traffic stimulus presented to the fish in aquaria is not completely identical to the noise experienced by fish in the environment. Distortions of the stimulus would occur at higher frequencies that are well beyond the documented auditory thresholds for *C*. *venusta*; the playback used in this study was a reliable representation of traffic noise in the environment.

While some fish have the opportunity to alter habitat use to avoid anthropogenic noise, the movement of fishes inhabiting small streams is restricted, including species of *Cyprinella* [36, 37]. Indeed, traffic noise propagated from a bridge can disrupt the natural soundscape of a stream up to a distance of over 12, 000 m [15]. Watersheds in urbanized regions may, therefore, provide few refuges from noise. As a result, populations of this species unable to avoid traffic noise may have degraded hearing for extended periods. Morning commuter traffic would be the most problematic as this species spawns early in the day [38]. As traffic noise is predominantly low frequency, it effectively masks the acoustic signals of *C*. *venusta* [15]. Masking, combined with decreased auditory sensitivity and elevated glucocorticoids, could have negative fitness consequences for *C*. *venusta* and other soniferous otophysans exposed to heavy traffic levels in the environment.

## Supporting Information

S1 Fig Traffic Noise Characteristics. (A) Oscillogram of traffic noise used in study. (B) Sonogram of traffic noise (1.95 Hz resolution).
(TIF)

S1 Table Stress Data. The weights and cortisol values for the traffic and control treatment for each test fish.
(DOCX)

S2 Table Auditory Thresholds. Auditory thresholds for fish exposed to either the traffic or control treatment. Thresholds are presented as dB re 1 μPa.
(DOCX)
