# Supplementary figures and images for: Stress and Auditory Responses of the Otophysan Fish, Cyprinella venusta, to Road Traffic Noise
Source: PLoS One. 2015 Sep 23;10(9):e0137290. doi: 10.1371/journal.pone.0137290 (PMC4580447; doi:10.1371/journal.pone.0137290)

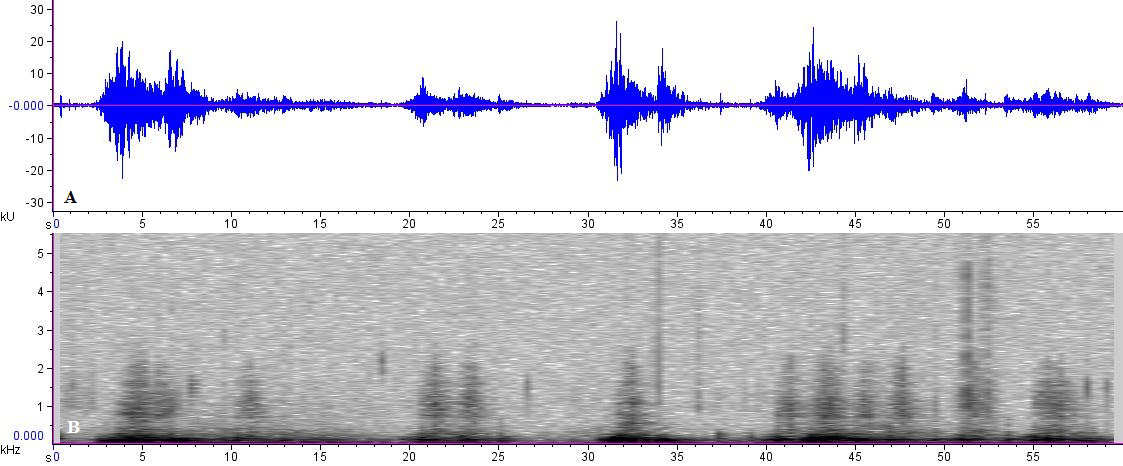

Supplement: S1 Fig — (A) Oscillogram of traffic noise used in study. (B) Sonogram of traffic noise (1.95 Hz resolution). (TIF) [file pone.0137290.s001.tif]
