# Supplementary material for: Stress and Auditory Responses of the Otophysan Fish, Cyprinella venusta, to Road Traffic Noise
Source: PLoS One. 2015 Sep 23;10(9):e0137290. doi: 10.1371/journal.pone.0137290 (PMC4580447; doi:10.1371/journal.pone.0137290)
Supplement: S1 Table — The weights and cortisol values for the traffic and control treatment for each test fish. (DOCX) [file pone.0137290.s002.docx]

**Table S1. Stress Data.**

| **Fish Weight (g)** | **Cortisol (ng/g): Traffic** | **Cortisol (ng/g): Control** |
| --- | --- | --- |
| 4.2 | 9.7 | 4.2 |
| 6.1 | 3.4 | 3.4 |
| 7.3 | 15.9 | 10.1 |
| 5.3 | 4.6 | 2.5 |
| 5.4 | 2.9 | 3.3 |
| 6.0 | 3.0 | 2.3 |
| 6.1 | 17.3 | 13.9 |

The weights and cortisol values for the traffic and control treatment for each test fish.
