# Supplementary material for: Stress and Auditory Responses of the Otophysan Fish, Cyprinella venusta, to Road Traffic Noise
Source: PLoS One. 2015 Sep 23;10(9):e0137290. doi: 10.1371/journal.pone.0137290 (PMC4580447; doi:10.1371/journal.pone.0137290)
Supplement: S2 Table — Auditory thresholds for fish exposed to either the traffic or control treatment. Thresholds are presented as dB re 1 μPa. (DOCX) [file pone.0137290.s003.docx]

**Table S2. Auditory Thresholds.**

| **Frequency (Hz)** | **Control Thresholds (dB re 1 µPa)** | | | | | **Traffic Thresholds (dB re 1 µPa)** | | | | |
| --- | --- | --- | --- | --- | --- | --- | --- | --- | --- | --- |
| **100** | 95 | 90 | 90 | 100 | 95 | 90 | 100 | 100 | 90 | 95 |
| **200** | 90 | 95 | 90 | 95 | 95 | 100 | 100 | 100 | 90 | 95 |
| **300** | 85 | 85 | 85 | 85 | 85 | 100 | 90 | 90 | 85 | 95 |
| **400** | 80 | 90 | 80 | 80 | 80 | 105 | 90 | 90 | 85 | 85 |
| **600** | 85 | 100 | 90 | 85 | 85 | 100 | 90 | 90 | 85 | 95 |
| **800** | 85 | 105 | 95 | 95 | 95 | 100 | 95 | 95 | 90 | 100 |
| **1,000** | 95 | 105 | 100 | 100 | 95 | 100 | 105 | 105 | 95 | 110 |

Auditory thresholds for each fish exposed to either the traffic or control treatment. Thresholds are presented as dB re 1 µPa.
